# XMU-MP-1 attenuates osteoarthritis via inhibiting cartilage degradation and chondrocyte apoptosis

**DOI:** 10.3389/fbioe.2022.998077

**Published:** 2022-09-19

**Authors:** Xue Hao, Jing Zhao, Liyuan Jia, Ting He, Huanbo Wang, Jing Fan, Yating Yang, Fei Su, Qingda Lu, Chao Zheng, Liu Yang, Qiang Jie

**Affiliations:** ^1^ Pediatric Orthopaedic Hospital, Honghui Hospital, Xi’an Jiaotong University, Xi’an, China; ^2^ Research Center for Skeletal Developmental Deformity and Injury Repair, School of Life Science and Medicine, Northwest University, Xi’an, China; ^3^ Clinincal Research Center for Pediatric Skeletal Deformity and Injury of Shaanxi Province, Xi’an, China; ^4^ College of Life Sciences, Northwest University, Xi’an, China; ^5^ Medical Research Institute, Northwestern Polytechnical University, Xi’an, China; ^6^ Institute of Orthopedic Surgery, Xijing Hospital, Air Force Medical University, Xi’an, China

**Keywords:** osteoarthritis, XMU-MP-1, Hippo signaling, chondrocyte, cartilage

## Abstract

Osteoarthritis (OA) is the most prevalent type of degenerative joint disease; it is reported to be associated with inflammatory responses, chondrocyte apoptosis, and cartilage degeneration. XMU-MP-1 is a selective MST1/2 inhibitor which activates the downstream effector YAP and promotes cell growth. It has displayed excellent benefits in mouse intestinal repair, as well as liver repair and regeneration. However, the effects of XMU-MP-1 on OA remain unclear. In this study, we investigated the therapeutic role of XMU-MP-1 on interleukin-1β (IL-1β)-induced inflammation in mice chondrocytes and the destabilization of the medial meniscus surgery (DMM)-induced OA model. In chondrocytes, treatment with XMU-MP-1 elevated the matrix metalloproteinases (*Mmp3*, *Mmp13*) and decreased the extracellular matrix (*Col2*, *Acan*) induced by IL-1β. Moreover, XMU-MP-1 strongly inhibited IL-1β-induced chondrocyte apoptosis and significantly promoted chondrocyte proliferation. Furthermore, XMU-MP-1 demonstrated a protective and therapeutic influence on the mouse OA model. These findings indicate that XMU-MP-1 may have a protective effect on cartilage degradation and may be a new potential therapeutic option for OA.

## Introduction

Osteoarthritis (OA) is the most common joint disease, affecting more than 240 million persons worldwide (Global Burden of Disease Study 2013 Collaborators, 2015). Patients with OA live life with severe joint pain, stiffness, and significantly reduced mobility, leading to decreased productivity and life quality, as well as there being an increased economic burden to patients and society (Litwic et al., 2013). However, current treatments for OA include pain amelioration and eventual joint replacement for end-stage disease, and lack an effective disease-modifying approach (Cao et al., 2020). The discovery of new treatment strategies for OA is urgently needed.

OA is a disorder disease involving the whole joint, with pathologic changes in cartilage, bone, synovium, ligament, muscle, and periarticular fat, among which the progressive destruction of articular cartilage is one of the main pathological manifestations (Sharma, 2021). The articular cartilage is a flexible tissue composed of extracellular matrix and chondrocytes; it provides an optimal surface to enable movements in the joint, prevents friction between the bones, and facilitates the transmission of loading to the underlying bone. Chondrocytes are the only cells in cartilage, synthesizing extracellular matrix to maintain cartilage homeostasis and thus preserving the structural and functional integrity of the cartilage (He Y. et al., 2020). During OA pathogenesis, risk factors such as aging and aberrant mechanical stress stimuli result in altered chondrocyte functioning in cartilage maintenance, and an imbalance of chondrocyte anabolism and catabolism (Hunter and Bierma-Zeinstra, 2019). Additionally, an increased level of apoptosis was observed in OA cartilage, resulting in decreased living chondrocytes and enhanced extracellular matrix degradation (Hwang and Kim, 2015). Therefore, an approach that integrates chondrocyte anabolism-catabolism homeostasis maintenance and chondrocyte apoptosis reduction provides a clue for a promising OA treatment.

Hippo signaling is an evolutionarily conserved pathway that plays a pivotal role in organ-size control by balancing cell proliferation and death; it consists of a core kinase cascade and downstream transcriptional effector (Yin and Zhang, 2011). Ste20-like kinases 1/2 (MST1/2) are the key components of the Hippo signaling pathway. The kinase cascade is composed of MST1/2, SAV1, LATS1/2, and MOB1A/B which conducts cellular signals to subsequently phosphorylate Yes-associated protein (YAP) and sequester YAP in the cytoplasm (Wu et al., 2003; Huang et al., 2005; Pan, 2007). When Hippo signaling is inactive, YAP translocates into nuclei, forming a complex with TEA domain transcription factor (TEADs) to promote the transcription of pro-proliferation and pro-survival genes (Wu et al., 2008; Zhang et al., 2008). Furthermore, recent studies have found that Hippo-YAP signaling plays an important role in chondrocyte maintenance and cartilage degeneration (Deng et al., 2018; Fu et al., 2019; Li et al., 2022). YAP alleviates senescence via up-regulation of FOXD1 (Fu et al., 2019) and attenuates NF-κB signaling by direct interaction with TAK1 in regulating matrix-degrading enzyme expression and cartilage degradation in OA pathogenesis (Deng et al., 2018). Considering the promoting effects of YAP on cell proliferation, the modulation of Hippo-YAP signaling activity may present a viable strategy for treating OA.

The small molecular compound XMU-MP-1 is a potent and selective inhibitor of MST1/2 that activates the downstream effector YAP, promoted cell growth and augmenting liver and intestinal repair and regeneration (Fuqin et al., 2016). However, the effects of XMU-MP-1 in OA pathogenesis remain unclear.

In the present study, we identified XMU-MP-1 as a compound with effects of anti-cartilage degeneration using *in vitro* and *in vivo* OA models. In cultured chondrocytes, a supplement of XMU-MP-1 rescued the elevation of catabolic factors, and the decrease of extracellular matrix synthesis induced by IL-1β treatment. Moreover, XMU-MP-1 strongly inhibited IL-1β-induced chondrocyte apoptosis and significantly promoted chondrocyte proliferation. Furthermore, XMU-MP-1 exhibited a protective and therapeutic influence on mice OA models. The mechanism study uncovered the crucial role of YAP in XMU-MP-1 protective effects in OA pathogenesis. In summary, these findings indicate that XMU-MP-1 may exert protective effects on cartilage degradation and may be a new potential therapeutic option for OA.

## Materials and methods

### Animals

All animal work performed in this study was approved by the Ethics in Animal Research Committee of the Air Force Medical University. In accordance with the standards for animal housing, mice were group-housed at 23°C–25°C with a 12-hour light/dark cycle and allowed free access to water and standard laboratory pellets.

To induce OA, male mice 8 weeks of age were subjected to DMM surgery or sham surgery at the right knee as described previously (Glasson et al., 2007). Briefly, in DMM surgery, the joint capsule was opened immediately after anesthesia, and the medial meniscotibial ligament was cut to destabilize the meniscus without damaging other tissues. In sham surgery, the joint capsule was opened in the same fashion but without any further damage.

For administration in mice, XMU-MP-1 was dissolved in DMSO (stock concentration, 5 mg/ml), diluted in corn oil by 1:10 before injection. Six weeks after DMM surgery, the mice received treatment intraperitoneally every other day for 2 weeks with XMU-MP-1 (1 mg/kg). Solution of 10% DMSO with 90% corn oil was used as a negative control. Mice were euthanized 2 months after surgery.

### Histology analyses

Mouse joints were fixed with 4% paraformaldehyde overnight, decalcified with 10% EDTA for 7 days, embedded in O.C.T. compound, and sectioned using a frozen slicer. The sections were stained with haematoxylin and eosin (H&E) and Safranin O/Fast Green to analyze phenotypic changes within the knee joint. Images were captured with an Olympus microscope. The articular cartilage destruction was quantified using the established Osteoarthritis Research Society International (OARSI) scoring system (score: 0–6) (Pritzker et al., 2006).

### Immunofluorescence

Immunostaining was performed according to the standard protocol.

For immunofluorescence analysis of the cartilage, frozen sections were incubated with antibodies specific for YAP (1:100, 14074; Cell Signaling), TAZ (1:100, 83669; Cell Signaling), MST1 (1:100, 14946; Cell Signaling), Collagen II (1:100, MA5-13026; ThermoFisher), and MMP13 (1:100, 18165-1-AP; proteintech) overnight at 4°C and then incubated with Cy3-conjugated secondary antibody (1:1000, BA1031, BA1032; BOSTER) for 1 h.

For immunofluorescence analysis of the primary chondrocytes, cells were fixed with 4% paraformaldehyde for 10 min, then incubated in 0.3% Triton X-100 for 15 min, blocked by blocking buffer (1% bovine serum albumin in PBS) for 15 min, and incubated with antibodies specific for YAP (1:100, 14074; Cell Signaling) and Collagen II (1:100, MA5-13026; ThermoFisher) and were then incubated with Cy3-conjugated secondary antibody (1:1000, BA1031, BA1032; BOSTER) for 1 h.

Images were captured with a laser-scanning confocal microscope (OLYMPUS FV1000) using the FV10-ASW 4.2 Viewer (OLYMPUS) and analyzed using ImageJ software.

### Primary chondrocyte extract, culture and treatment

For primary culture of chondrocytes, rib cartilage was isolated from P0-3 pups and digested with collagenase D solution (3 mg/ml, Roche) for 1 h at 37°C under 5% CO_2_ in a thermal incubator. Tissue fragments were agitated several times to detach soft tissues and then digested for 4 h at 37°C. The digestion solution was retrieved, filtered through a 45 μm cell strainer and then centrifuged for 5 min at 1,000 rpm. The pellet was re-suspended with DMEM (Gbico) supplemented with 10% fetal bovine serum (Gbico), 50 U per ml penicillin and 0.05 mg per ml streptomycin. The chondrocytes were seeded at a density of 2 × 10^4^ cells per cm^2^ and cultured under sterile conditions at 37°C under 5% CO_2_. Only the first passage cells were used for experiments.

For experiments, the chondrocytes were treated with 1 ng/ml recombinant murine IL-1β (P10749; PEPROTECH) for 48 h in the presence or absence of XMU-MP-1 (S8334; Selleck).

### siRNA transfection

Duplex siRNA targeting *Yap* was purchased from GenePharma. When cells grew to 60%–70% density, siRNAs were transfected into cells *in vitro* with Lipofectamine RNAiMAX (Invitrogen) in accordance with the manufacturer’s instructions, followed by treatment of IL-1β and/or XMU-MP-1 for 48 h.

The sequences were as follows: siRNA-control: UUC​UCC​GAA​CGU​GUC​ACG​UTT; siRNA-*Yap*: GGU​CAA​AGA​UAC​UUC​UUA​ATT.

### Micromass culture and alcian blue staining

For micromass culture, chondrocytes (4 × 10^5^ cells in 20 μl) were dropped into round droplets in wells of a four-well chamber slide (SPL-30404; Bio-Arrow), waiting for attachment at 37°C for 4 h, then supplemented with 500 μl cell culture medium and cultured for 3 days. After treatment of IL-1β and/or XMU-MP-1 for 48 h, the cells were subjected to Alcian blue staining. The micromass cultures were washed with PBS three times, then fixed at room temperature for 15 min in 4% paraformaldehyde, and stained with Alcian blue solution (1.0% Alcian blue in 0.1 N HCl) for 4 h; ddH2O washes were performed until no color was visible in the supernatant. Images were acquired with a Zeiss microscope.

### RNA extraction and quantitative RT-PCR

Total RNA was extracted from cultured chondrocytes with MiniBEST Universal RNA Extraction Kit (9767; Takara) according to manufacturer’s instructions, and the cDNAs were synthesized using ReverTra Ace qPCR RT Master Mix with gDNA Remover (FSQ-301; TOYOBO). Real-time PCR was performed using the SYBR Green Real-time PCR Master Mix (QPK-201; TOYOBO) reagent with the BioRAD CFX96 System. Results were repeated for three independent biological replicates. *Gapdh* was used as a normalized control. The sequence of PCR primers used in the study were listed in Supplementary Table S1.

### EdU staining

To access the proliferation of chondrocytes, EdU staining assay was utilized using BeyoClick™ EdU Cell Proliferation Kit with Alexa Fluor 555 (C0075S; Beyotime). Images were captured with a laser-scanning confocal microscope (OLYMPUS FV1000) using the FV10-ASW 4.2 Viewer (OLYMPUS), then analyzed using ImageJ software. Five random visions per sample were chosen to capture the images.

### Flow cytometry analysis

The apoptosis of the chondrocytes was evaluated by flow cytometry analysis. The cells were stained using the FITC Annexin V Apoptosis Detection Kit I (556547; BD Pharmingen), and then analyzed through a fluorescence-activated cell sorting (FACS) flow cytometer (Coulter-XL; Beckman Coulter).

### Statistical analysis

Statistical analysis was performed with GraphPad Prism software, and the results were given as mean ± SEM. Differences between experimental groups were assessed using the unpaired two-tailed Student’s t-test or one-way ANOVA with Bonferroni post hoc test: * is *p* < 0.05, ** is *p* < 0.01, *** is *p* < 0.001, **** is *p* < 0.0001, and “ns” is no significance with *p* > 0.05.

## Results

### Hippo signaling is hyper-activated in OA chondrocytes

**FIGURE 1:**
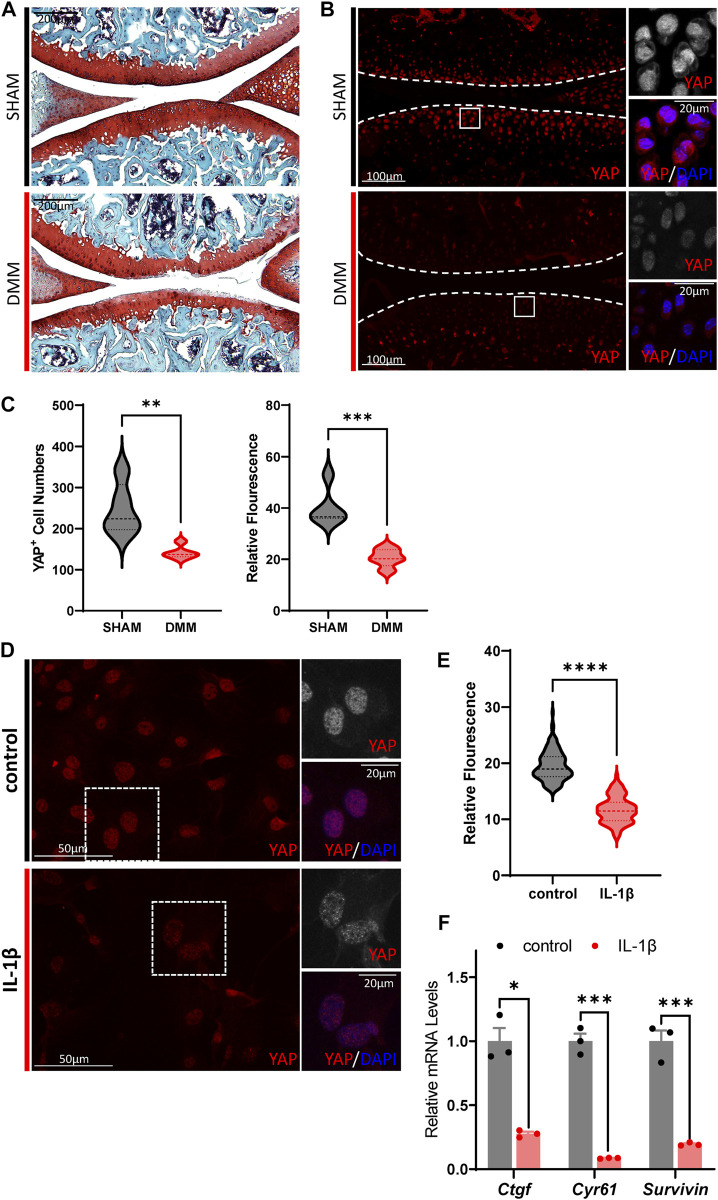
Hippo signaling is hyper-activated in OA chondrocytes. **(A)** Representative images of Safranin O/Fast Green staining of knee joint sections from wild-type mice which underwent sham or DMM surgery for 8 weeks. **(B)** Representative images of knee joint sections from wild-type mice which underwent sham or DMM surgery for 8 weeks. Sections were immunostained with antibody against YAP (red or gray) and DAPI (nuclei, blue). Panels on the right are high magnifications of boxed regions in the left panels. The dashed lines marked the edge of cartilage. **(C)** Statistical analysis of the YAP^+^ chondrocytes in articular cartilage in B. Panel on the left is the quantification of YAP^+^ chondrocytes numbers per cartilage. Panel on the right is the quantification of YAP relative fluorescence in YAP^+^ chondrocytes. *n* = 5, 5. **(D)** Representative images of cultured primary chondrocytes treated with IL-1β at 1 ng/ml, or not. Cells were immunostained with antibody against YAP (red or gray) and DAPI (nuclei, blue). Panels on the right are high magnifications of the boxed regions in the left panels. **(E)** Statistical analysis of relative fluorescence of YAP in D. *n* = 3, 3. **(F)** Relative mRNA levels of YAP target genes *Ctgf*, *Cyr61* and *Survivin* in primary chondrocytes treated with IL-1β or not. All data are presented as mean ± SEM. *p < 0.05, **p < 0.01, ***p < 0.001.

Since much research on the role of Hippo-YAP signaling in OA pathogenesis has had no clear conclusion (Zhong et al., 2013; Karystinou et al., 2015; Deng et al., 2018; Goto et al., 2018; Fu et al., 2019; Gong et al., 2019; Zhang Q. et al., 2019; Zhang X. et al., 2019; He J. et al., 2020; Li et al., 2022), we first investigated the Hippo signaling activity change in OA cartilage chondrocytes. DMM surgery was used to recapitulate the degenerative condition of articular cartilage in mice joint. Two months after surgery, the articular cartilage degeneration could be observed by Safranin O/Fast Green staining, in which the expression of YAP and TAZ, the key mediator of Hippo signaling, showed a dramatic reduction in the chondrocytes (Figures 1A–C; Supplementary Figures S1A,B). Furthermore, the expression of Hippo core kinase MST1, which regulates YAP upstream, was observed to be increased in the OA cartilage chondrocytes (Supplementary Figures S1C,D). Taken together, these results indicate hyper-activation of Hippo signaling in OA cartilage chondrocytes.

Next, to validate the results of hyper-activation of Hippo signaling in OA cartilage, treatment of murine IL-1β was conducted on cultured primary chondrocytes to induce inflammation and simulate the OA phenotype. As shown in Figures 1D,E, YAP expression decreased dramatically upon IL-1β stimulation in cultured murine primary chondrocytes. The transcription levels of canonical YAP targets, *Ctgf*, *Cyr61*, and *Survivin*, were observed to be downregulated in IL-1β treated cells, demonstrating the suppressed activity of YAP. Altogether, these results show that Hippo signaling is hyper-activated in OA chondrocytes, which in turn inhibits YAP activity and reduces YAP expression.

### Protective effects of XMU-MP-1 on ECM degradation and chondrogenesis

**FIGURE 2:**
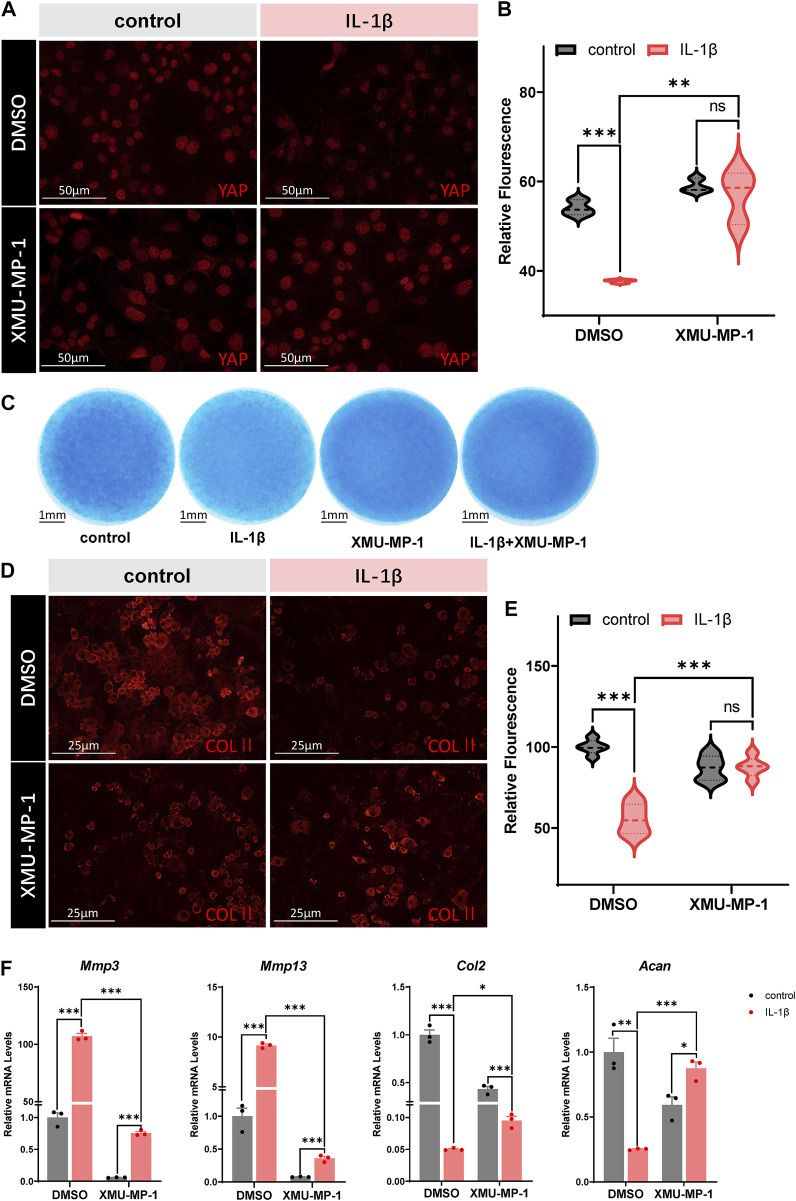
Protective effects of XMU-MP-1 on ECM degradation and chondrogenesis. **(A)** Representative images of cultured primary chondrocytes treated with IL-1β or/and XMU-MP-1 for 48 h. Cells were immunostained with antibody against YAP (red). **(B)** Statistical analysis of relative fluorescence of YAP in cultured chondrocytes in A. *n* = 3, 3, 3, 3. **(C)** Representative images of chondrocyte micromass cultures treated with IL-1β or/and XMU-MP-1 for 48 h. The micromass cultures were subjected to Alcian blue staining. **(D)** Representative images of cultured primary chondrocytes treated with IL-1β or/and XMU-MP-1 for 48 h. Cells were immunostained with antibody against COLII (red). **(E)** Statistical analysis of relative fluorescence of COLII in D. n = 5, 5, 5, 5. **(F)** Relative mRNA levels of *Mmp3*, *Mmp13, Col2*, and *Acan* in primary chondrocytes treated with IL-1β or/and XMU-MP-1 for 48 h. All data are presented as mean ± SEM. ns *p* > 0.05, **p < 0.01, ***p < 0.001.

Based on our results, we propose that restoring YAP in OA chondrocytes might have chondroprotective effects against arthritis. The small molecular compound XMU-MP-1 was identified as a selective inhibitor of MST1/2 that enhanced downstream YAP activation and exhibited *in vivo* efficacy in liver and intestinal repair and regeneration (Fuqin et al., 2016). Thus, here comes up with our hypothesis that XMU-MP-1 may be a potentially effective drug against OA. We first investigated whether XMU-MP-1was capable of restoring YAP in IL-1β treated chondrocytes. As shown in Figures 2A,B, co-treatment of XMU-MP-1 rescued the downregulation of YAP expression induced by IL-1β. Micromass culture was then employed to monitor the protective effects of XMU-MP-1 on chondrogenesis. Alcian Blue staining of the micromass cultures showed that exposure to IL-1β resulted in impaired chondrogenesis, while supplementing with XMU-MP-1 recovered the damaged chondrogenesis (Figure 2C). Immunofluorescence staining of type II collagen in chondrocytes treated by IL-1β or/and XMU-MP-1 consistently suggested that degradation of extracellular matrix induced by IL-1β treatment was inhibited by XMU-MP-1 (Figures 2D,E). Damaged chondrogenesis usually includes reduced cartilage matrix synthesis and enhanced cartilage matrix degradation (Moon-Chang et al., 2019). Accordingly, IL-1β treatment led to increased expression of matrix degrading enzymes and decreased expression of ECM components. We examined expression of matrix degrading enzymes *Mmp3* and *Mmp13*, and ECM components *Collagen II* and *Aggrecan* at mRNA levels to address the role of XMU-MP-1 in cartilage matrix maintenance. As shown in Figure 2F, compared with the IL-1β-treated group, co-treatment with XMU-MP-1 restored decrease of *Collagen II* and *Aggrecan* expression, accompanied with the inhibition of elevated *Mmp3* and *Mmp13* expression. Furthermore, different dosages (5 μM or 25 μM) of XMU-MP-1 resulted in similar effects in catabolic factor suppression, while various conditions of treatment suggested that XMU-MP-1 functions in a time-dependent manner (Supplementary Figures S2A,B). Taken together, these results indicate that treatment of XMU-MP-1 results in the activation of YAP in OA chondrocytes and serves chondroprotective functions.

### XMU-MP-1 facilitates proliferation and suppresses apoptosis of OA chondrocytes

**FIGURE 3:**
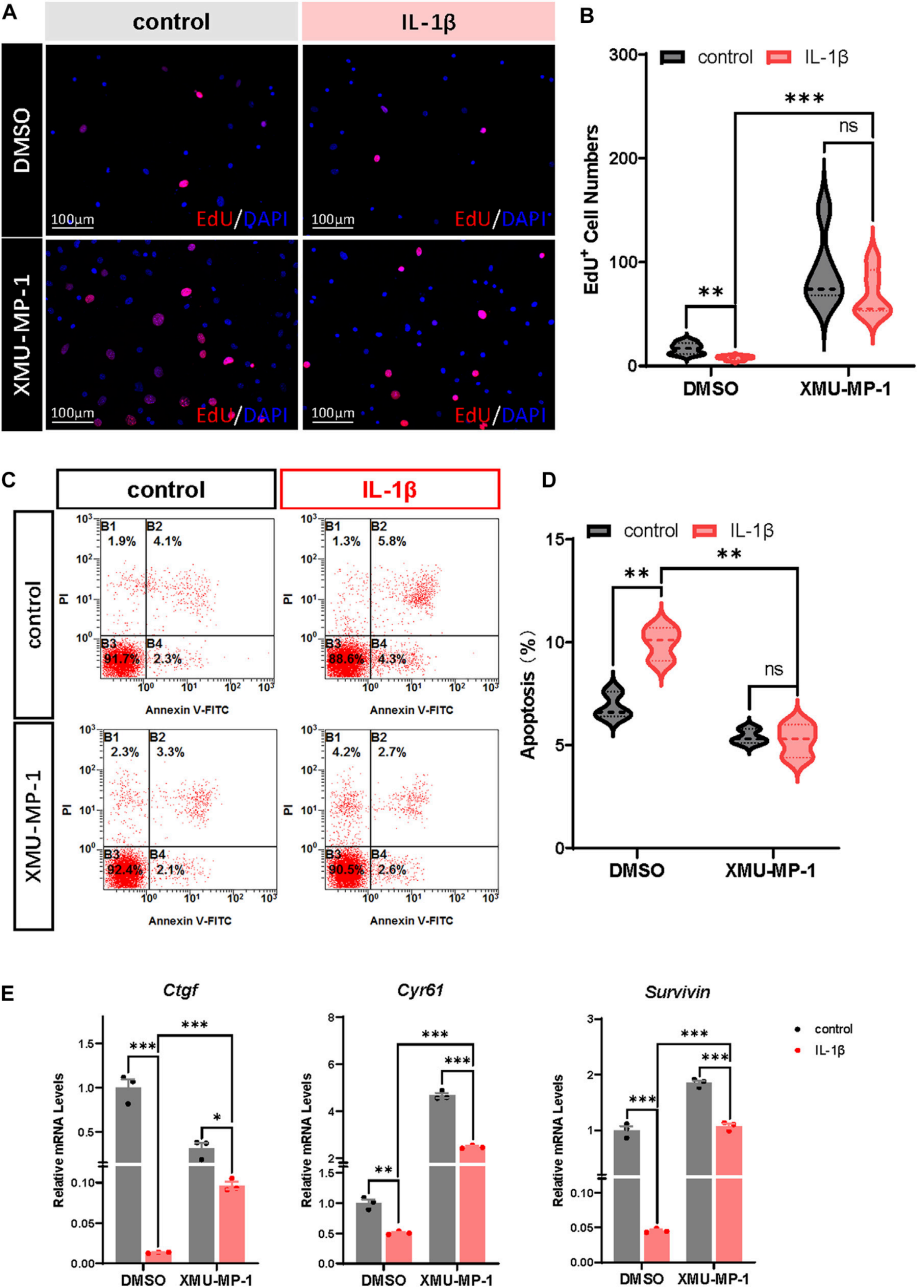
XMU-MP-1 facilitates proliferation and suppresses apoptosis of OA chondrocytes. **(A)** Representative images of EdU labeling chondrocytes treated with IL-1β or/and XMU-MP-1 for 48 h. EdU (5-ethynyl-2′-deoxyuridine) was added during cell culture and subsequently stained with a fluorescent dye (red) and DAPI (nuclei, blue). **(B)** Statistical analysis of the EdU^+^ chondrocytes numbers in B. **(C)** Representative flow cytometry graphs of cultured primary chondrocytes stained with Annexin V-FITC and propidium iodide (PI) treated with IL-1β or/and XMU-MP-1 for 48 h. **(D)** Statistical analysis of apoptotic cells in B. **(E)** Relative mRNA levels of YAP target genes *Ctgf*, *Cyr61*, and *Survivin* in chondrocytes treated with IL-1β or/and XMU-MP-1 for 48 h. All data are presented as mean ± SEM. ns *p* > 0.05, *p < 0.05, **p < 0.01, ***p < 0.001.

Previous studies have demonstrated that OA chondrocytes exhibit reduced proliferation and increased apoptosis. Combined with the role of Hippo-YAP signaling in regulating cell proliferation and apoptosis, we surmised that XMU-MP-1 offers unique advantages in manipulating OA chondrocyte proliferation and apoptosis. EdU labeling experiments were carried out to explore the function of XMU-MP-1 in chondrocyte proliferation regulation. As shown in Figures 3A,B, suppressed proliferation of chondrocytes induced by IL-1β treatment was rescued by co-treatment of XMU-MP-1. Additionally, Flow cytometry analysis showed that XMU-MP-1 restored increased cell apoptosis induced by IL-1β in chondrocytes (Figures 3C,D). Further mechanism study revealed that XMU-MP-1 treatment promoted the expression of *Ctgf*, *Cyr61*, and *Survivin*, which are responsible for cell proliferation and apoptosis regulation (Figure 3E). As described above, these results suggest that XMU-MP-1 facilitates proliferation and suppresses apoptosis of OA chondrocytes.

### XMU-MP-1 attenuates OA pathogenesis

**FIGURE 4:**
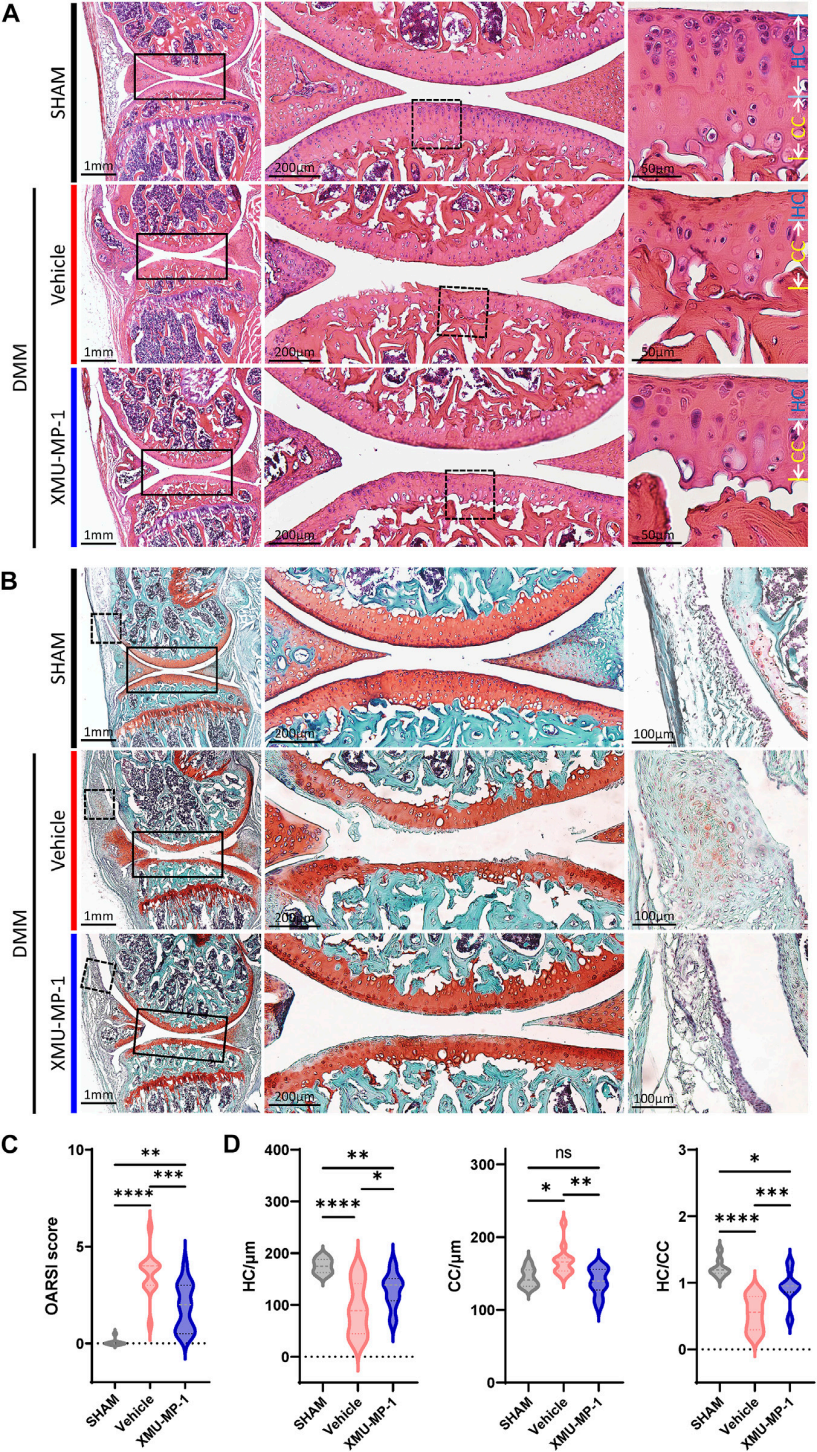
XMU-MP-1 alleviates articular cartilage degeneration during OA process. **(A)** Representative images of HE staining of knee joint sections from mice of indicated groups. SHAM: Mice underwent sham surgery. Vehicle: Mice undergoing DMM surgery were intraperitoneally injected with vehicle. XMU-MP-1: Mice undergoing DMM surgery were intraperitoneally injected with XMU-MP-1. Panels in the middle are high magnifications of boxed regions in the left panels; panels on the right are high magnifications of boxed regions in the middle panels. **(B)** Representative images of Safranin O/Fast Green staining of knee joint sections from mice of indicated groups. SHAM: Mice underwent sham surgery. Vehicle: Mice undergoing DMM surgery were intraperitoneally injected with vehicle. XMU-MP-1: Mice undergoing DMM surgery were intraperitoneally injected with XMU-MP-1. Panels in the middle are the high magnification of the solid box-like area in the left panels, panels on the right are the high magnification of the dotted box-like area in the left panels. **(C)** Statistical analysis of OARSI scores in B. *n* = 8, 12, 14. **(D)** Statistical analysis of thickness of hyaline cartilage (HC), calcified cartilage (CC), and ratio of HC to the CC in A. *n* = 8, 12, 14. All data are presented as mean ± SEM. ns *p* > 0.05, *p < 0.05, **p < 0.01, ***p < 0.001, ****p < 0.0001.

**FIGURE 5:**
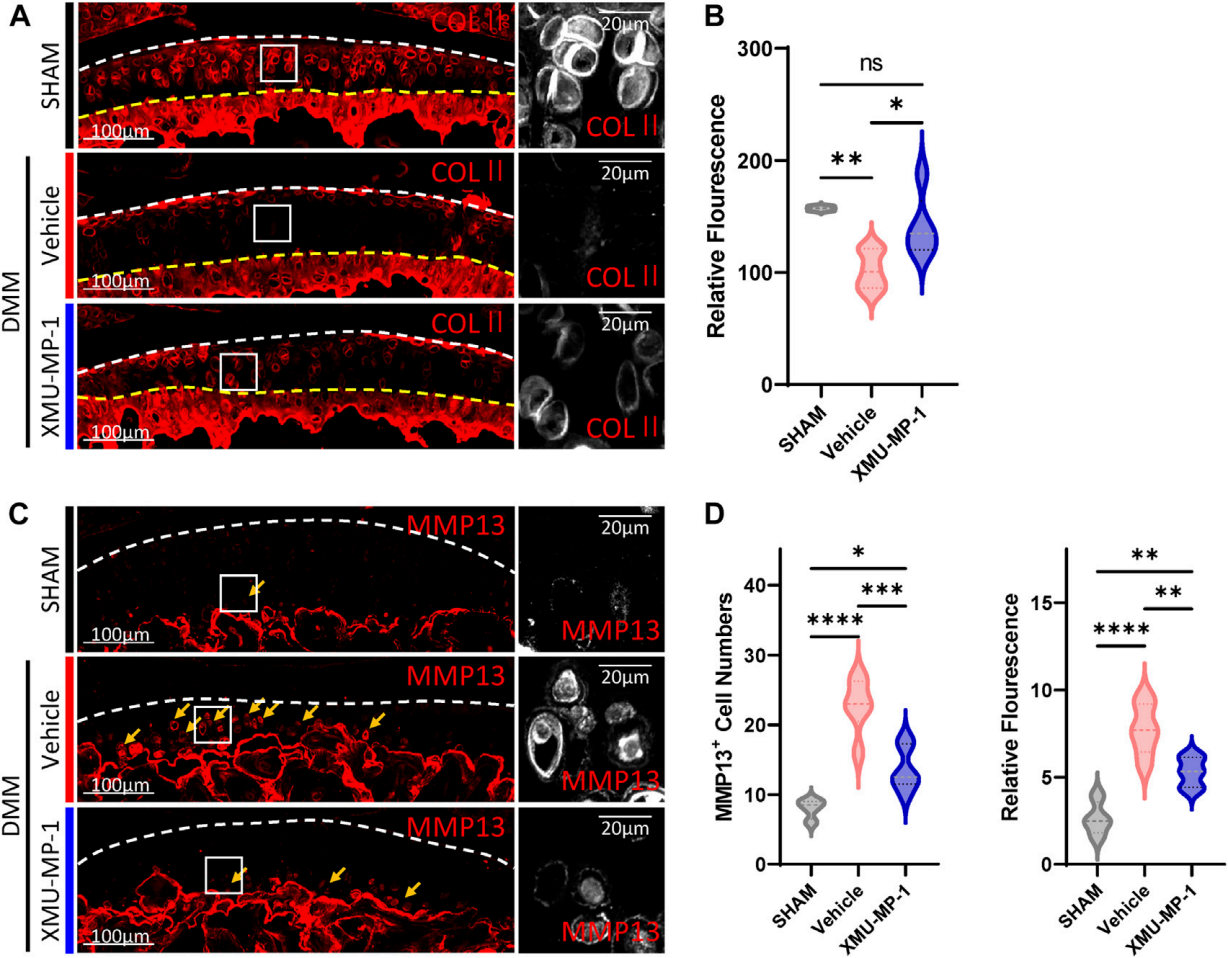
XMU-MP-1 attenuates articular cartilage degradation during OA pathogenesis. **(A)** Representative images of knee joint sections of mice from indicated groups. SHAM: Mice underwent sham surgery. Vehicle: Mice undergoing DMM surgery were intraperitoneally injected with vehicle. XMU-MP-1: Mice undergoing DMM surgery were intraperitoneally injected with XMU-MP-1. Sections were immunostained with antibody against COLⅡ (red or gray). Panels on the right are high magnifications of boxed regions in the left panels. The white dashed lines marked the edge of cartilage. The yellow dashed lines marked the tidemark. **(B)** Statistical analysis of the relative fluorescence of COLⅡ in hyaline cartilages in A. *n* = 3, 6, 5. **(C)** Representative images of knee joint sections of mice from indicated groups. SHAM: Mice underwent sham surgery. Vehicle: Mice undergoing DMM surgery were intraperitoneally injected with vehicle. XMU-MP-1: Mice undergoing DMM surgery were intraperitoneally injected with XMU-MP-1. Sections were immunostained with antibody against MMP13 (red or gray). Panels on the right are high magnifications of boxed regions in the left panels. The dashed lines marked the edge of cartilage. The yellow arrows mark the MMP13^+^ chondrocytes in calcified cartilage. **(D)** Statistical analysis of the MMP13^+^ chondrocytes numbers in articular cartilage in C. Panel on the left is quantification of MMP13^+^ chondrocytes numbers per cartilage. Panel on the right is the quantification of relative fluorescence in MMP13^+^ chondrocytes. *n* = 3, 6, 6. All data are presented as mean ± SEM. *p < 0.05, **p < 0.01, ***p < 0.001, ****p < 0.0001.

Following to the above results, we explored the role of XMU-MP-1 in OA pathogenesis *in vivo*. DMM surgery was carried out to successfully establish the mouse OA model that articular cartilage degeneration could be observed in Safranin O/Fast Green staining, as well as a decrease in the ratio of thickness of hyaline cartilage to calcified cartilage (HC/CC) (Figure 4). As previously described, XMU-MP-1 exhibited favorable pharmacokinetics with a half-life of 1.2 h, and a minimal dose of 1 mg/kg via intraperitoneal injection (Fuqin et al., 2016). Therefore, we administered XMU-MP-1 via intraperitoneal injection with a frequency of once every other day from 6 weeks after DMM surgery for two more weeks (Supplementary Figure S3A). To address the concern that systemic administration of XMU-MP-1 may cause unexpected side effects, the weight curve of mice along the treatment was monitored. No detectable difference in weight between control and treatment groups indicated no obvious harm to health (Supplementary Figure S3B). To evaluate the protective effects of XMU-MP-1 on cartilage degeneration, haematoxylin and eosin (H&E) and Safranin O/Fast Green staining were performed to evaluate the histomorphological differences. The OARSI score, based on the histological features of OA progression, provides an overall assessment of cartilage OA status, including depth of progression and the extent of surface area affected (Wenzel, et al., 2016). Compared with the vehicle group, administration of XMU-MP-1 suppressed erosion on the articular cartilage surfaces, synovial thickening, and restored the OARSI score and HC/CC ratio (Figure 4). Mechanistically, decrease of ECM components (type II collagen) and elevated expression of matrix degradation enzyme MMP13 in OA cartilage induced by DMM surgery were also blocked by XMU-MP-1 (Figure 5), indicating decreased matrix degradation—consistent with the results from cultured chondrocytes. Taken together, these results demonstrate that XMU-MP-1 attenuates articular cartilage degeneration during OA pathogenesis.

### YAP plays a crucial role in XMU-MP-1 chondroprotective function during OA pathogenesis

**FIGURE 6:**
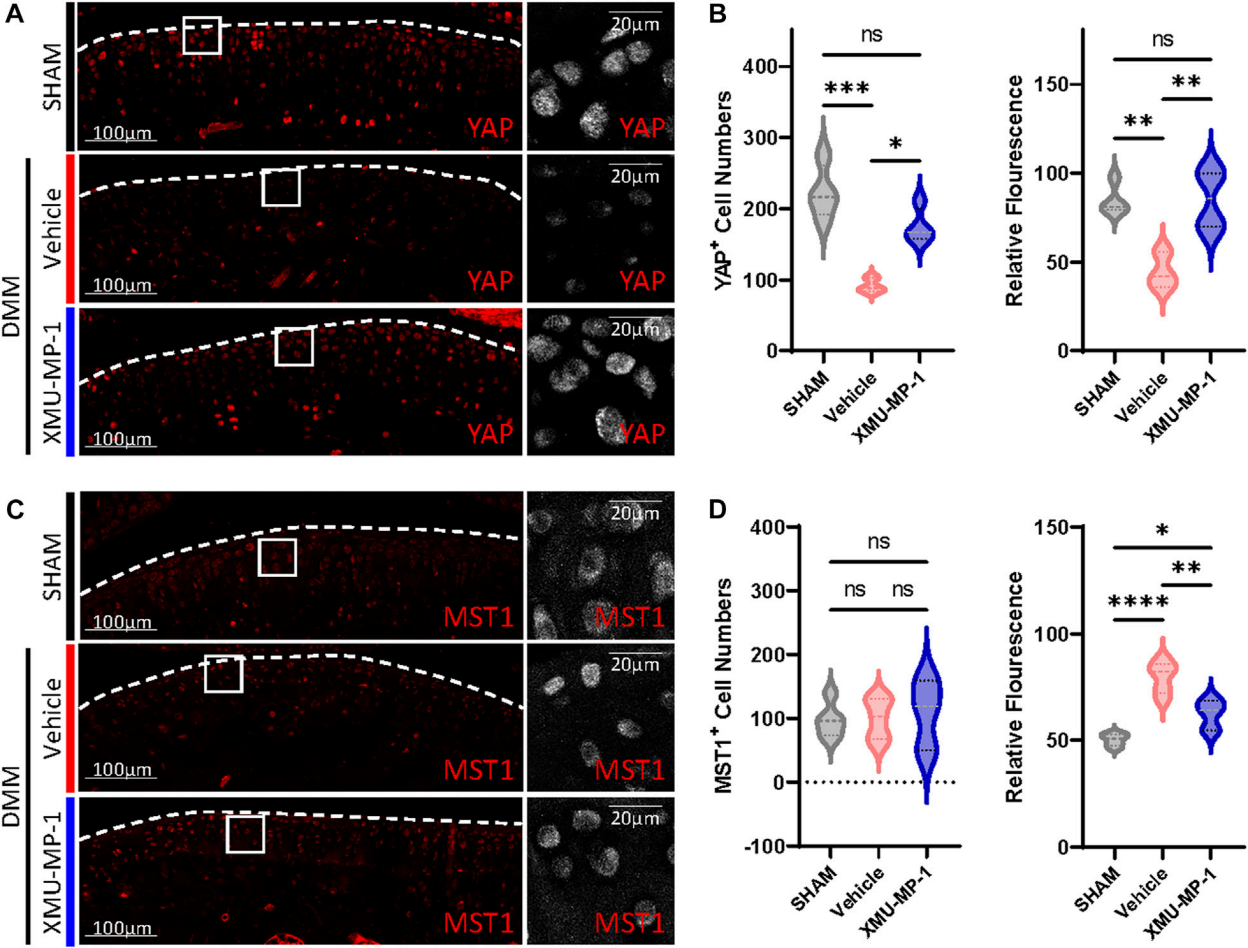
XMU-MP-1 attenuates OA pathogenesis via YAP. Representative images of knee joint sections of mice from indicated groups. SHAM: Mice underwent sham surgery. Vehicle: Mice undergoing DMM surgery were intraperitoneally injected with vehicle. XMU-MP-1: Mice undergoing DMM surgery were intraperitoneally injected with XMU-MP-1. Sections were immunostained with antibody against YAP (red or gray) **(A)** and MST1 (red or gray) **(C)**. **(B)** Statistical analysis of the YAP^+^ chondrocytes numbers in articular cartilage in A. Panel on the left is quantification of YAP^+^ chondrocytes numbers per cartilage. Panel on the right is the quantification of relative fluorescence in YAP^+^ chondrocytes. *n* = 4, 3, 4. **(D)** Statistical analysis of the MST1^+^ chondrocytes numbers in articular cartilage in C. Panel on the left is quantification of MST1^+^ chondrocytes numbers per cartilage. Panel on the right is the quantification of relative fluorescence in MST1^+^ chondrocytes. *n* = 5, 5, 3. All data are presented as mean ± SEM. *p < 0.05, **p < 0.01, ***p < 0.001, ****p < 0.0001.

**FIGURE 7:**
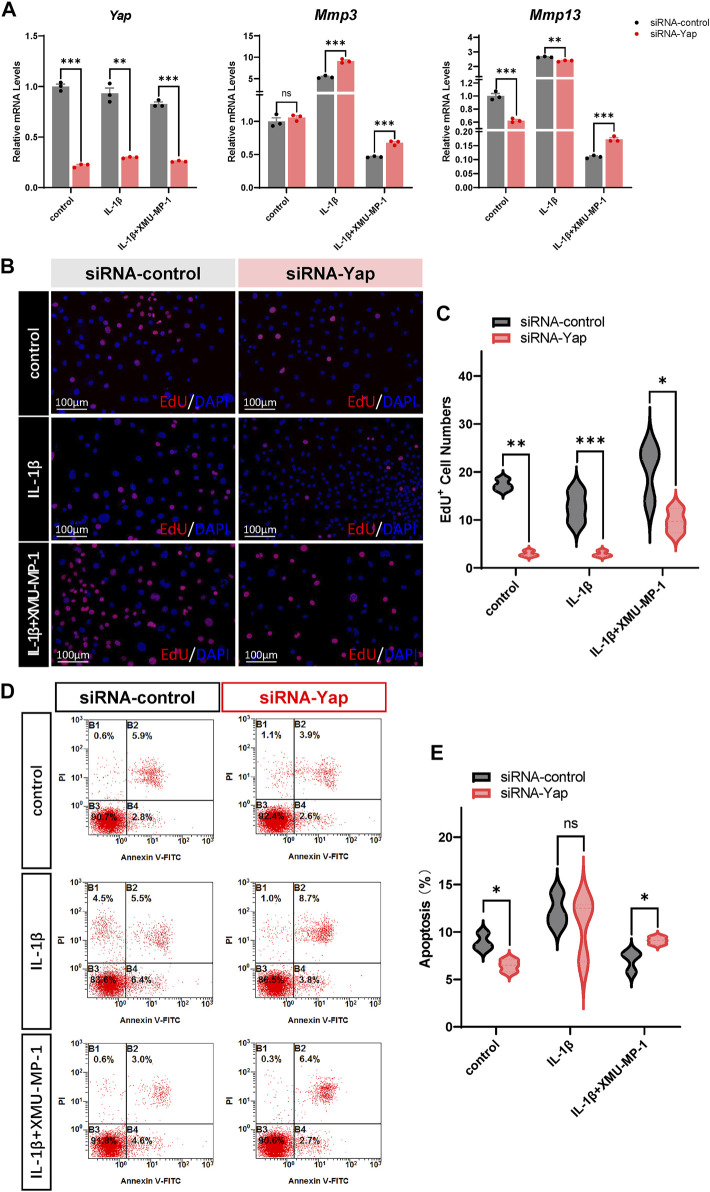
YAP plays a crucial role in XMU-MP-1 chondroprotective function during OA pathogenesis. **(A)** Relative mRNA levels of *Yap*, *Mmp3,* and *Mmp13* in primary chondrocytes transfected with the control or *Yap* siRNA, treated with IL-1β or/and XMU-MP-1. **(B)** Representative images of EdU labeling chondrocytes transfected with the control or *Yap* siRNA, treated with IL-1β or/and XMU-MP-1. Cells stained with a fluorescent dye (red) and DAPI (nuclei, blue). **(C)** Statistical analysis of the EdU^+^ chondrocytes numbers in B. **(D)** Representative flow cytometry graphs in primary chondrocytes transfected with the control or *Yap* siRNA, treated with IL-1β or/and XMU-MP-1. **(E)** Statistical analysis of apoptotic cells in D. All data are presented as mean ± SEM. ns *p* > 0.05, *p < 0.05.

We next investigated the underlying mechanism of the protective effects of XMU-MP-1 on cartilage degeneration during the OA process. Firstly, the expression of YAP and MST1 in cartilage chondrocytes from the vehicle or XMU-MP-1 treatment groups were examined. As expected, administration of XMU-MP-1 restored the increase of MST1 and decrease of YAP in cartilage chondrocytes (Figure 6). Since we noticed that the expression of YAP changed more obviously than MST1, we hypothesized—accompanied by the present view that YAP is downstream effector of MST1/2 in Hippo signaling—that YAP plays a crucial role in XMU-MP-1 chondroprotective functions. *Yap* siRNA was transfected in chondrocytes to knockdown YAP. As shown in Figure 7A, inhibition of *Mmp3* and *Mmp13* by XMU-MP-1 treatment were partially de-repressed in *Yap* depleted chondrocytes. EdU labeling experiments showed suppressed cell proliferation by *Yap* knockdown upon XMU-MP-1 stimulation (Figures 7B,C). Additionally, Flow cytometry analysis showed that decreased apoptosis by XMU-MP-1 treatment was blocked by YAP depletion (Figures 7D,E). Altogether, these results demonstrate that XMU-MP-1 attenuates cartilage degeneration via regulating matrix degradation and chondrocyte proliferation-apoptosis balance in a YAP-dependent manner.

## Discussion

The articular cartilage is a highly differentiated tissue lacking blood vessels for nutrition supply, leading to irreversibility in cartilage degradation and difficulty in the effective disease-modified therapy of OA. An approach combining anabolism-catabolism homeostasis maintenance and chondrocyte apoptosis reduction may promise an OA treatment. In this study, we identified compound XMU-MP-1 as a potential therapeutic agent to moderate OA cartilage degeneration. Cultured primary chondrocytes were exposed to IL-1β to induce inflammation and simulate OA phenotype *in vitro*, resulting in an imbalance of chondrocyte anabolism and catabolism and increased apoptosis, which were restored by co-treatment of XMU-MP-1. Additionally, administration of XMU-MP-1 in mice via intraperitoneal injection alleviated DMM-induced cartilage degeneration. Further mechanism study revealed that XMU-MP-1 exerts chondroprotective effects in a YAP-dependent manner. As well as the research on promoting the effects of XMU-MP-1 in liver and intestine recovery and regeneration, our work demonstrates for the first time that XMU-MP-1 may exert a protective effect on cartilage degradation and serve as a new potential therapeutic option for OA.

Chondrocytes are the only cells sparsely distributed in the extracellular matrix in cartilage. Thus, their survival is important for proper cartilage matrix maintenance; compromising chondrocyte function and survival will lead to articular cartilage failure. It has been previously reported that, in OA cartilage, 18%–21% of chondrocytes showed apoptotic features, compared with 2%–5% in normal cartilage (Heraud et al., 2000). Chondrocyte death leads to the failure of cartilage homeostasis maintenance (Hwang and Kim, 2015). It is notable that the Hippo signaling pathway exerts a critical role in cell proliferation and apoptosis by modulating the transcription of target genes (Zheng and Pan, 2019). MST1/2 inhibitor XMU-MP-1 takes advantage of Hippo-YAP signaling in cell proliferation and apoptosis regulation. In the present work, we revealed the anti-apoptotic and pro-proliferation function of XMU-MP-1 on OA chondrocyte in a YAP-dependent manner.

As well as apoptosis, impaired ECM catabolism also contributes to OA. Catabolism of the cartilage ECM is partially mediated by a variety of degradative enzymes, including matrix metalloproteinases (MMPs) and ADAMTS (a disintegrin and metalloproteinase with thrombospondin motifs), by which both collagen fibrils and proteoglycans are degraded (JA et al., 2001; Glasson et al., 2005). However, clinical trials that directly target catabolic enzymes met with limited success and have resulted in side effects including musculoskeletal pain and inflammation (Cao et al., 2020). In recent decades, numerous mechanism studies regulating cartilage degradation and chondrocyte maintenance have been performed to explore therapeutic targets. Several signaling pathways—such as NF-κB signaling, Wnt signaling, and Hippo-YAP signaling—have been proved critical for OA pathogenesis (Moon-Chang et al., 2019; Wang et al., 2019; Zarka et al., 2021). However, current studies investigating the role of Hippo-YAP signaling in the OA process mainly focus on YAP, rather than on other core components of Hippo signaling, in modulating chondrocyte maintenance and cartilage degradation (Deng et al., 2018; Zhang X. et al., 2019; Fu et al., 2019). In the present study, knockdown of *Yap* by siRNA in chondrocytes partially abolished the XMU-MP-1 protective effects on extracellular matrix degradation induced by IL-1β treatment; this indicates the possibility of YAP-independent mechanisms. Several studies have unveiled the engagement of MST1/2 in the regulation of the innate immune independence of the canonical effectors YAP/TAZ. For instance, the induction of mitochondria trafficking and reactive oxygen species (ROS) is impaired in *Mst1/2* deficient phagocytes after bacterial infection (Geng et al., 2015). Together with these findings, our mechanism study results raise the possibility of MST1/2 in cartilage matrix catabolism via non-canonical Hippo signaling cascade.

On the other hand, the role of YAP in OA is still under debate. Besides the anti-inflammatory role of YAP mediated by interaction with TAK1 in OA cartilage chondrocytes, YAP is also demonstrated to serve as a mechanotransducer of chondrocytes on chondrocyte-phenotype maintenance (Deng et al., 2018; Zhang X. et al., 2019). This raises the concern that activation of YAP by XMU-MP-1 treatment may lead to chondrocyte dedifferentiation and failure in cartilage matrix maintenance. Given the fact that XMU-MP-1 is a selective inhibitor of MST1/2, one of numerous kinases undertaking transduction of the cellular signaling via phosphorylation of downstream YAP/TAZ, activation of YAP by XMU-MP-1 is quite mild—no such phenomenon of ECM destruction or chondrocyte dedifferentiation in XMU-MP-1 treated micromass cultures was observed in the present study (Figure 2C).

As the sole population existing in cartilage, the poor reproducing capacity of chondrocytes impairs cartilage regeneration. Our work demonstrated that XMU-MP-1 promotes chondrocyte proliferation, which will benefit future bio-material research on cartilage regeneration. Together with its role in anti-apoptosis and cartilage matrix maintenance, XMU-MP-1 may serve as a promising therapeutic option for OA.

## Data Availability

The original contributions presented in the study are included in the article/Supplementary Material. Further inquiries can be directed to the corresponding author.

## References

[B1] Cao P. Yamin L. Yujin T. Changhai D. David J H. (2020). Pharmacotherapy for knee osteoarthritis: Current and emerging therapies. Expert Opin. Pharmacother. 21, 797. 10.1080/14656566.2020.1732924 32100600

[B2] Deng Y. Lu J. Li W. Wu A. Zhang X. Tong W. (2018). Reciprocal inhibition of YAP/TAZ and NF-κB regulates osteoarthritic cartilage degradation. Nat. Commun. 9, 4564. 10.1038/s41467-018-07022-2 30385786 PMC6212432

[B3] Fu L. Hu Y. Song M. Liu Z. Zhang W. Yu F.-X. (2019). Up-regulation of foxd1 By Yap alleviates senescence and osteoarthritis. PLoS Biol. 17, E3000201. 10.1371/journal.pbio.3000201 30933975 PMC6459557

[B4] Fuqin F. Zhixiang H. Lu-Ku K. Qinghua C. Quan Y. Shihao Z. (2016). Pharmacological targeting of kinases Mst1 and Mst2 augments tissue repair and regeneration. Sci. Transl. Med. 8, 352ra108. 10.1126/scitranslmed.aaf2304 27535619

[B5] Geng J. Sun X. Wang P. Zhang S. Wang X. Wu H. (2015). Kinases Mst1 and Mst2 positively regulate phagocytic induction of reactive oxygen species and bactericidal activity. Nat. Immunol. 16, 1142–1152. 10.1038/ni.3268 26414765 PMC4618176

[B6] Glasson S. S. Askew R. Sheppard B. Carito B. Blanchet T. Ma H.-L. (2005). Deletion of active Adamts5 prevents cartilage degradation in A murine model of osteoarthritis. Nature 434, 644–648. 10.1038/nature03369 15800624

[B7] Glasson S. S. Blanchet T. J. Morris E. A. (2007). The surgical destabilization of the medial meniscus (dmm) model of osteoarthritis in the 129/svev mouse. Osteoarthr. And Cartil. 15, 1061–1069. 10.1016/j.joca.2007.03.006 17470400

[B8] Global Burden of Disease Study 2013 Collaborators (2015). Global, regional, and national incidence, prevalence, and years lived with disability for 301 acute and chronic diseases and injuries in 188 countries, 1990-2013: A systematic analysis for the global burden of disease study 2013. Lancet (London, Engl. 386, 743–800. 10.1016/S0140-6736(15)60692-4 PMC4561509 26063472

[B9] Gong Y. Li S.-J. Liu R. Zhan J.-F. Tan C. Fang Y.-F. (2019). Inhibition of Yap with sirna prevents cartilage degradation and ameliorates osteoarthritis development. J. Mol. Med. 97, 103–114. 10.1007/s00109-018-1705-y 30465058

[B10] Goto H. Nishio M. To Y. Oishi T. Miyachi Y. Maehama T. (2018). Loss of Mob1a/B in mice results in chondrodysplasia due to yap1/taz-tead-dependent repression of Sox9. Development 145, dev159244. 10.1242/dev.159244 29511023

[B11] He J. Su X. Xie W. (2020a). Mir-582-3p alleviates osteoarthritis progression by targeting Yap1. Mol. Immunol. 128, 258–267. 10.1016/j.molimm.2020.10.022 33190006

[B12] He Y. Li Z. Alexander P. Ocasio-Nieves B. Yocum L. Lin H. (2020b). Pathogenesis of osteoarthritis: Risk factors, regulatory pathways in chondrocytes, and experimental models. Biology 9, 194. 10.3390/biology9080194 PMC7464998 32751156

[B13] Heraud F. Heraud A. Harmand M. F. (2000). Apoptosis in normal and osteoarthritic human articular cartilage. Ann. Rheum. Dis. 59, 959–965. 10.1136/ard.59.12.959 11087699 PMC1753049

[B14] Huang J. Wu S. Barrera J. Matthews K. Pan D. (2005). The Hippo signaling pathway coordinately regulates cell proliferation and apoptosis by inactivating yorkie, the Drosophila homolog of Yap. Cell 122, 421–434. 10.1016/j.cell.2005.06.007 16096061

[B15] Hunter D. J. Bierma-Zeinstra S. (2019). Osteoarthritis. Lancet 393, 1745–1759. 10.1016/s0140-6736(19)30417-9 31034380

[B16] Hwang H. S. Kim H. A. (2015). Chondrocyte apoptosis in the pathogenesis of osteoarthritis. Int. J. Mol. Sci. 16, 26035–26054. 10.3390/ijms161125943 26528972 PMC4661802

[B17] Ja M. Mp V. Ce B. (2001). Il-1 induces collagenase-3 (Mmp-13) promoter activity in stably transfected chondrocytic cells: Requirement for runx-2 and activation by P38 mapk and jnk pathways. Nucleic Acids Res. 29, 4361–4372. 10.1093/nar/29.21.4361 11691923 PMC60184

[B18] Karystinou A. Roelofs A. J. Neve A. Cantatore F. P. Wackerhage H. De Bari C. (2015). Yes-associated protein (Yap) is A negative regulator of chondrogenesis in mesenchymal stem cells. Arthritis Res. Ther. 17, 147. 10.1186/s13075-015-0639-9 26025096 PMC4449558

[B19] Li H.-N. Bai-Ming J. Hua Z. Le-Le L. Meng-Yuan L. Xiu-Juan Z. (2022). Yap plays A protective role in T-2 toxin-induced inhibition of chondrocyte proliferation and matrix degradation. Toxicon 215, 49–56. 10.1016/j.toxicon.2022.06.005 35697129

[B20] Litwic A. Edwards M. H. Dennison E. M. Cooper C. (2013). Epidemiology and burden of osteoarthritis. Br. Med. Bull. 105, 185–199. 10.1093/bmb/lds038 23337796 PMC3690438

[B21] Moon-Chang C. Jiwon J. Jonggwan P. Hee Kyoung K. Yoonkyung P. (2019). Nf-κb signaling pathways in osteoarthritic cartilage destruction. Cells 8, 734. 10.3390/cells8070734 PMC6678954 31319599

[B22] Pan D. (2007). Hippo signaling in organ size control. Genes Dev. 21, 886–897. 10.1101/gad.1536007 17437995

[B23] Pritzker K. P. H. Gay S. Jimenez S. A. Ostergaard K. Pelletier J. P. Revell P. A. (2006). Osteoarthritis cartilage histopathology: Grading and staging. Osteoarthr. And Cartil. 14, 1–2. 10.1016/j.joca.2005.08.015 16242352

[B24] Sharma L. (2021). Osteoarthritis of the knee. N. Engl. J. Med. Overseas. Ed. 384, 51–59. 10.1056/nejmcp1903768 33406330

[B25] Wang Y. Fan X. Xing L. Tian F. (2019). Wnt signaling: A promising target for osteoarthritis therapy. Cell Commun. Signal. 17, 97. 10.1186/s12964-019-0411-x 31420042 PMC6697957

[B26] Wenzel W. Giorgio P. Susannah L G. Suzanne A M. Reinhard W. Friedrich B. (2016). Oarsi osteoarthritis cartilage histopathology assessment system: A biomechanical evaluation in the human knee. J. Orthop. Res. 34, 135–140. 10.1002/jor.23010 26250350

[B27] Wu S. Huang J. Dong J. Pan D. (2003). Hippo encodes A ste-20 family protein kinase that restricts cell proliferation and promotes apoptosis in conjunction with salvador and warts. Cell 114, 445–456. 10.1016/s0092-8674(03)00549-x 12941273

[B28] Wu S. Liu Y. Zheng Y. Dong J. Pan D. (2008). The tead/tef family protein scalloped mediates transcriptional output of the Hippo growth-regulatory pathway. Dev. Cell 14, 388–398. 10.1016/j.devcel.2008.01.007 18258486

[B29] Yin M. Zhang L. (2011). Hippo signaling: A hub of growth control, tumor suppression and pluripotency maintenance. J. Genet. Genomics 38, 471–481. 10.1016/j.jgg.2011.09.009 22035868

[B30] Zarka M. Haÿ E. Cohen-Solal M. (2021). Yap/taz in bone and cartilage biology. Front. Cell Dev. Biol. 9, 788773. 10.3389/fcell.2021.788773 35059398 PMC8764375

[B31] Zhang L. Ren F. Zhang Q. Chen Y. Wang B. Jiang J. (2008). The tead/tef family of transcription factor scalloped mediates Hippo signaling in organ size control. Dev. Cell 14, 377–387. 10.1016/j.devcel.2008.01.006 18258485 PMC2292673

[B32] Zhang Q. Fang X. Zhao W. Liang Q. (2019a). The transcriptional coactivator Yap1 is overexpressed in osteoarthritis and promotes its progression by interacting with beclin-1. Gene 689, 210–219. 10.1016/j.gene.2018.11.068 30496783

[B33] Zhang X. Cai D. Zhou F. Yu J. Wu X. Yu D. (2019b). Targeting downstream subcellular Yap activity as A function of matrix stiffness with verteporfin-encapsulated chitosan microsphere attenuates osteoarthritis. Biomaterials 232, 119724. 10.1016/j.biomaterials.2019.119724 31918221

[B34] Zheng Y. Pan D. (2019). The Hippo signaling pathway in development and disease. Dev. Cell 50, 264–282. 10.1016/j.devcel.2019.06.003 31386861 PMC6748048

[B35] Zhong W. Li Y. Li L. Zhang W. Wang S. Zheng X. (2013). Yap-mediated regulation of the chondrogenic phenotype in response to matrix elasticity. J. Mol. Histol. 44, 587–595. 10.1007/s10735-013-9502-y 23543231

