# Supplementary material for: XMU-MP-1 attenuates osteoarthritis via inhibiting cartilage degradation and chondrocyte apoptosis
Source: Front Bioeng Biotechnol. 2022 Sep 19;10:998077. doi: 10.3389/fbioe.2022.998077 (PMC9527278; doi:10.3389/fbioe.2022.998077)
Supplement: Supplementary file 1 [file DataSheet1.PDF]

Supplementary Material

1 Supplementary Figures and Tables

1.1 Supplementary Figures

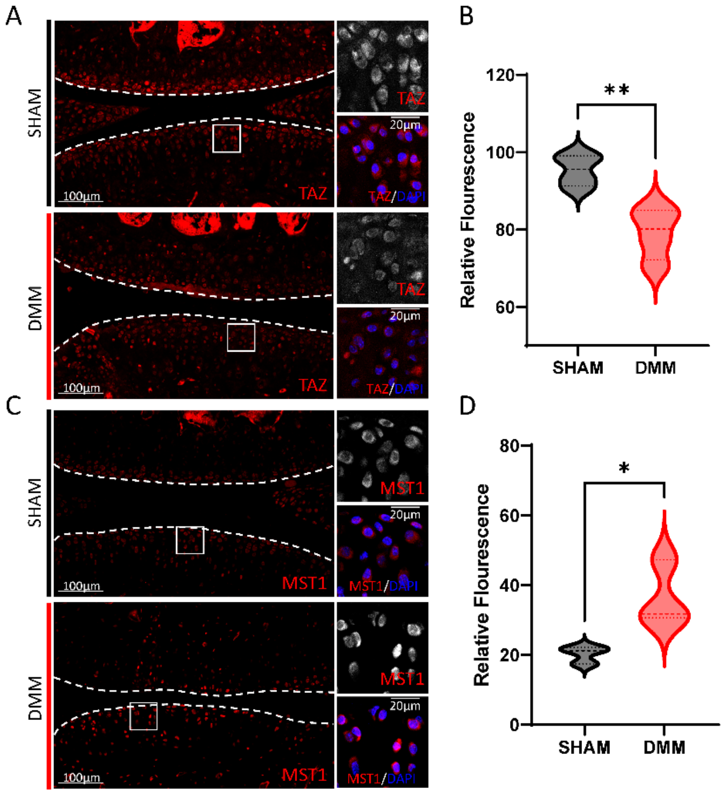

Supplementary Figure 1 Hippo signaling is hyper-activated in OA chondrocytes **(A)** Representative images of knee joint sections from wild-type mice underwent sham or DMM surgery for 8 weeks. Sections were immunostained with antibody against TAZ (red or gray) and DAPI (nuclei, blue). Panels on the right are high magnifications of boxed regions in the left panels. The dashed lines marked the edge of cartilage. **(B)** Statistical analysis of the TAZ^+ chondrocytes numbers in articular cartilage in A. n= 4, 4. **(C)** Representative images of knee joint sections from wild-type mice underwent sham or DMM surgery for 8 weeks. Sections were immunostained with antibody against MST1 (red or gray) and DAPI (nuclei, blue). Panels on the right are high magnifications of boxed regions in the left panels. The dashed lines marked the edge of cartilage. **(D)** Statistical analysis of the $MST1^+$ chondrocytes numbers in articular cartilage in C. n= 3, 3. All data are presented as mean ± SEM. * $p< 0.05$. ** $p< 0.01$.

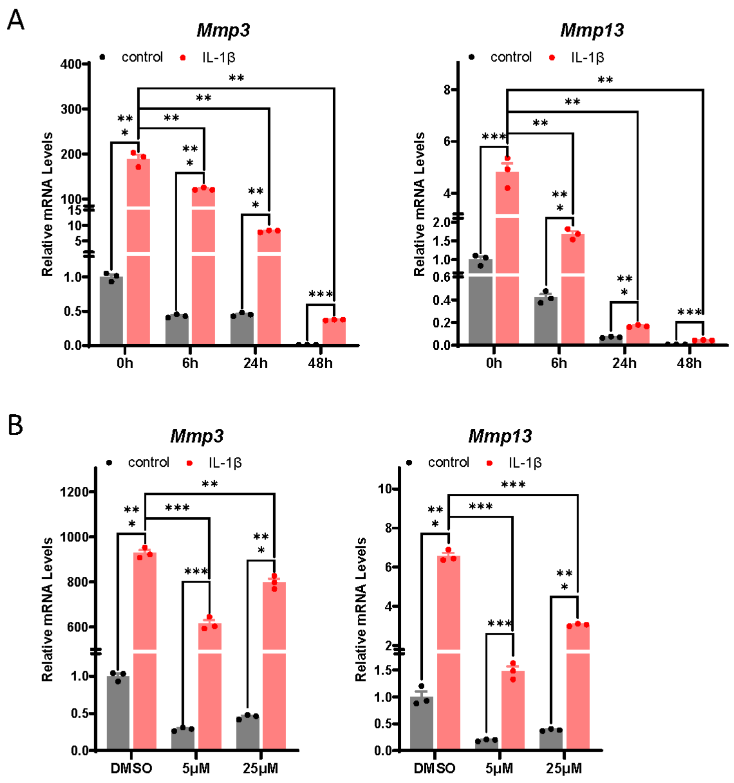

Supplementary Figure 2 Protective effects of XMU-MP-1 on ECM degradation **(A)** Relative mRNA levels of *Mmp3*, *Mmp13* in primary chondrocytes treated with IL-1β and co-treated with XMU-MP-1 for indicated time. **(B)** Relative mRNA levels of *Mmp3*, *Mmp13* in primary chondrocytes treated with IL-1β and co-treated with XMU-MP-1 for indicated dosage. All data are presented as mean ± SEM. ** p< 0.01, *** p< 0.001.

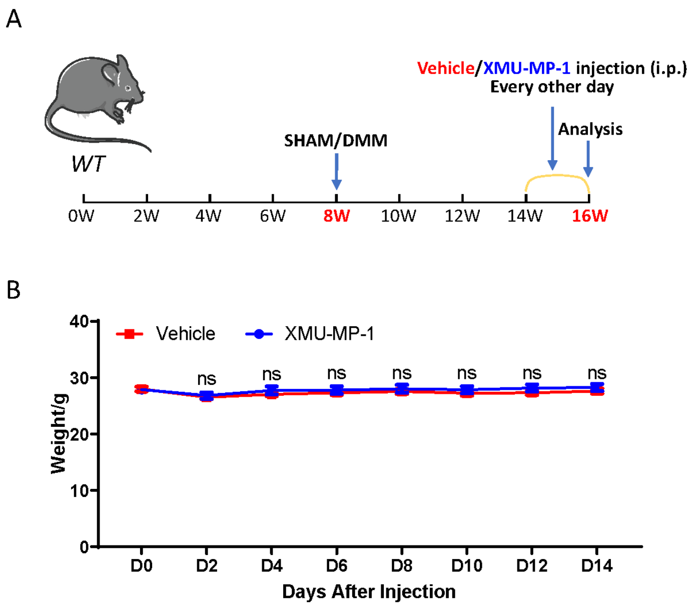

Supplementary Figure 3 XMU-MP-1 alleviates articular cartilage degeneration. (A) Schematic of XMU-MP-1 administration in mice. Male mice at 8-week-age received sham or DMM surgery. XMU-MP-1 is administrated via intraperitoneally injection once the other day from 6 weeks after the surgery for 2 weeks. Vehicle was used as control. The knee joints were analyzed 8 weeks after surgery. (B) Curves of body weight of mice from indicated group after intraperitoneal injection. Red: Vehicle; Blue: XMU-MP-1. n= 7, 6. All data are presented as mean ± SEM. ns p>0.05.

1.2 Supplementary Tables

Supplementary Table 1. Primer sequences of related genes.

Gene	Primer sequence
Gapdh-F	AGGTCGGTGTGAACGGATTTG
Gapdh-R	TGTAGACCATGTAGTTGAGGTCA
Ctgf-F	TCCGGACACCTAAAATCGCC
Ctgf-R	TTCATGATCTCGCCATCGGG
Cyr61-F	AGAGGCTTCCTGTCTTTGGC
Cyr61-R	CTCGTGTGGAGATGCCCAGTT
Survivin-F	AGAACAAAATTGCAAAGGAGACCA
Survivin-R	GGCATGTCACTCAGGTCCAA
Mmp3-F	TGGACAGAGGATGTCACTGGTA
Mmp3-R	GCCTTGGCTGAGTGGTAGAG
Mmp13-F	CTTCTTCTTGTTGAGCTGGACTC
Mmp13-R	CTGTGGAGGTCACTGTAGACT
Col2-F	CACCAAATTCCTGTTCAGCC
Col2-R	TGCACGAAACACACTGGTAAG
Acan-F	GGAGCGAGGCCATTTACAAC
Acan-R	CGTAGACAAGGTAGCCCACTTT
